# Dietary Macronutrient Composition Directs ChREBP Isoform Expression and Glucose Metabolism in Mice

**DOI:** 10.1371/journal.pone.0168797

**Published:** 2016-12-19

**Authors:** Tara Jois, Victor Howard, Kristina Youngs, Michael A. Cowley, Mark W. Sleeman

**Affiliations:** 1 Department of Physiology, Monash University, Clayton, Victoria, Australia; 2 Biomedicine Discovery Institute, Monash University, Clayton, Victoria, Australia; 3 Department of Biochemistry and Molecular Biology, Monash University, Clayton, Victoria, Australia; INRA, FRANCE

## Abstract

Carbohydrate response element binding protein (ChREBP) is a lipogenic transcription factor that is thought to be involved in the development of hepatic steatosis and insulin resistance. Increased ChREBP expression in liver results in increased hepatic steatosis, and the isoform ChREBPβ in adipose tissue can predict insulin sensitivity in obese humans. As ChREBP is activated by glucose, it was postulated that the composition of diet would regulate ChREBP isoform expression in metabolically relevant tissues.

We compared the effects of diets with high complex carbohydrate, high fat, or a normal chow on ChREBP expression and metabolic parameters in C57BL/6 mice. We found that diets high in fat decrease ChREBP expression in adipose tissue, but isocaloric diets high in carbohydrate have no effect. Interestingly, this decrease in adipose ChREBP was associated with increased inflammatory markers. In the same animals a high carbohydrate diet induced a robust increase in hepatic ChREBPβ expression (≈2-fold; p = 0.0002), but little detectable change in the more abundant ChREBPα transcript. This change was accompanied by increased expression of target genes liver pyruvate kinase (p<0.0001), acetyl-CoA carboxylase (p = 0.0191) and stearoyl-CoA desaturase-1 (p = 0.0045). This increase in ChREBP expression was associated with increased hepatic steatosis, despite no changes in body weight or body fat when compared to chow-fed mice. Unexpectedly, mice fed a high carbohydrate diet displayed enhanced sensitivity to exogenous insulin, despite having mild glucose intolerance and increased liver steatosis.

In summary, we have shown the composition of diet can selectively regulate ChREBP isoform expression in a tissue specific manner. Furthermore, we have shown a high complex carbohydrate diet selectively increases hepatic ChREBPβ expression, which associates with hepatic steatosis but not insulin resistance. In contrast, a high fat diet reduces adipose ChREBP, which associates with inflammation and insulin resistance.

## Introduction

Carbohydrate response element binding protein (ChREBP) is a transcription factor that regulates genes involved in glycolysis, gluconeogenesis and lipogenesis [1]. It has a key role in *de novo* lipogenesis in the liver, but is also expressed in various other tissues including white adipose tissue, brown adipose tissue, small intestine, kidney, and pancreas [2–5]. There are two isoforms of ChREBP, α and β, and the β isoform is thought to be more transcriptionally active [6]. ChREBP is activated by glucose and its metabolites (glucose-6-phosphate, xylulose 5-phosphate, fructose-2,6-bisphosphate) [7–11], and can be inhibited by polyunsaturated fatty acids and ketone bodies [12, 13].

ChREBP regulates liver *de novo* lipogenesis and there is strong evidence that it plays a leading role in the development of hepatic steatosis. There is increased expression of ChREBP in the liver in both obese animals and humans [14–17], and selective over-expression of ChREBP in the liver worsens hepatic steatosis in mice [18]. Furthermore, inhibition of ChREBP specifically in the liver of *ob/ob* mice results in decreased lipogenesis and hepatic steatosis [19]. The role of ChREBP in the development of insulin resistance is less clear however, as global ChREBP deletion in mice causes glucose intolerance and insulin resistance [3], yet ChREBP deletion in *ob/ob* mice results in improved glucose tolerance and insulin sensitivity [16, 19]. In humans, variants in the ChREBP gene, *MLXIPL*, have been associated with elevated plasma triglyceride levels and coronary artery disease [20–22], supporting a role for ChREBP in metabolic health. Regulation of ChREBP expression is not uniform across individual tissues, which is highlighted by the finding that obese individuals have increased expression in the liver but decreased levels in the adipose tissues [14, 15]. Furthermore, it has been proposed that increased adipose ChREBPβ expression, but not ChREBPα, correlates with insulin sensitivity in humans and conversely, a decreased expression in adipose tissue and increased expression in liver predicts insulin resistance [6, 14]. Considering the associations between ChREBP isoform mRNA expression and metabolic health, the transcriptional regulation of ChREBP isoforms warrants further investigation.

Although it is known certain metabolites can affect ChREBP cellular location and activity, less known is the influence of diets high in complex carbohydrates. Most studies to date have examined diets high in sugars [23–25], as high fat and high sugar consumption is thought to be the primary cause of diet-induced obesity in the Western world. Previous studies have shown the glycemic index of a meal influences postprandial glucose and insulin responses in humans [26]. Furthermore, high-glycemic index diets have been independently associated with increased risk of type 2 diabetes and coronary heart disease [27]. However, India has more cases of diabetes than anywhere else in the world despite their dairy, meat and sugar intake being within recommended levels [28, 29]. In fact, the major source of energy in Indian populations is refined carbohydrates such as white rice, and various studies have associated dietary carbohydrate consumption with insulin resistance and metabolic syndrome in this population [28, 30–32]. In this study we developed a high calorie, high complex-carbohydrate diet where the majority of energy is from the refined carbohydrate wheat starch (HCD, 18 MJ/kg: 70% carbohydrates, 21% protein, 9% lipids), as this arguably reflects the increased consumption of processed foods more accurately, and compared the effects of this diet to a high calorie, high fat diet (HFD, 19 MJ/kg: 36% carbohydrates, 21% protein, 43% lipids) both with equal contribution from sucrose. In this way we were able to examine how ChREBP was influenced by different dietary challenges and how changes in ChREBP were related to changes in metabolic health in mice.

It was found that not only did the composition of diet influence ChREBP expression in a tissue-specific manner, but also that the two ChREBP isoforms are differentially responsive to diet. Our data has shown a high-calorie high-complex carbohydrate diet selectively increases ChREBPβ expression in the liver, and this is associated with increased hepatic steatosis yet improved insulin sensitivity. In contrast, a high-fat diet selectively decreases ChREBP in adipose tissue and this is associated with increased inflammation and insulin resistance. This study provides insight into the complex regulation of ChREBP isoforms in metabolic tissues, adds further evidence to the notion that hepatic steatosis is not a causal factor in the development of insulin resistance, and highlights a novel role for ChREBP in diet-induced inflammation in obesity.

## Methods

### *In vivo* Diet Characterization

C57Bl6/J mice were acquired at 6 weeks of age from Monash Animal Services (MAS) institutional colony. Group housed animals were kept on a 12-hour light-dark cycle with free access to food and water. All conditions and experiments were reviewed and approved by Monash University Animal Ethics committee. Mice were placed on diet starting at 8 weeks of age and body weights were recorded twice a week. All diets were generated by Specialty Feeds (Glen Forrest, Western Australia). High fat diet (SF04-001, 45% calories from lipids) was based on Research Diets D12451. High carbohydrate diet (SF13-067, 70% calories from carbohydrates) was created using SF04-001 as a template with similar vitamin and mineral composition. A low fat diet (#12145, 4% calories from lipids) was used as a control chow in all experiments. See S1 File for detailed diet compositions. At experimental endpoint (after 6 or 12 weeks on diet) mice were anaesthetised by isoflurane inhalation, euthanized by cervical dislocation and tissues rapidly harvested for further analysis. All animals were sacrificed concomitantly at 10am and no animals died prior to experimental endpoint.

### Body composition

Body composition was assessed by dual-energy X-ray absorptiometry (DEXA) (Piximus, Lunar) every two weeks up to 12 weeks on diet.

### Glucose and insulin tolerance tests

To assess glucose tolerance, 18-hour fasted animals were dosed with a 3g/kg glucose solution P.O. and blood glucose was measured at 0, 30, 60, and 120 minutes via tail blood. To assess insulin tolerance, 4-hour fasted animals were dosed with 0.75U/kg insulin (Actrapid, Novo Nordisk) I.P. and blood glucose was measured at 0, 15, 30, 60, and 120 minutes via tail blood. Animals were evaluated for glucose and insulin tolerance every two weeks up to 12 weeks on diet.

### Indirect calorimetry

After 12 weeks on diet, mice were individually housed in metabolic chambers (CLAMS, Columbus Instruments) in order to assess metabolic activity. Mice were acclimated for 48 hours before data was collected. Mice had free access to food and water. Oxygen consumption, carbon dioxide production and ambulatory activity were measured, and RER and energy expenditure were calculated.

### Analytical procedures

Serum and plasma samples were obtained by retro-orbital venepuncture under isoflurane anaesthesia. Glucose stimulated insulin secretion (GSIS) was induced by dosing with a 3g/kg glucose solution and collecting blood after 30 minutes. Circulating insulin levels were assessed by sandwich ELISA (80-INSMSU-E01, ALPCO). Plasma resistin and PAI-1 were assessed by multiplexed bead assay (Bio-Plex Pro mouse diabetes assay, Bio-Rad) using the MAGPIX instrument (Luminex). Pancreatic insulin was obtained by alcohol/acid extraction [33]. Liver triglycerides were extracted via chloroform/ethanol method [34].

### Protein Analysis

Tissues for protein expression were flash frozen in liquid nitrogen after 12 weeks on diet. 20μg of tissue lysate was used for all Western blots. Insulin signaling was evaluated by Akt and phospho-Akt (Ser473) signaling (9272 & 4051, Cell Signaling). Quantification of bands was performed by densitometry via ImageJ (NIH).

### Histology

Livers were frozen in liquid nitrogen-cooled isopentane, then embedded in OCT (4583, Tissue-Tek). Frozen blocks were sectioned at 10μm, mounted and dried, and stained with Oil Red O and Haemotoxylin and Eosin.

### qPCR

RNA was extracted from liquid nitrogen flash-frozen samples and purified using a column-based RNEasy kit (74104, Qiagen). RNEasy Lipid Tissue kit (74804; QIAGEN) was used for RNA extraction of adipose samples. Extracted RNA was quantified and checked for purity using an Implen NanoPhotometer (Implen GmbH, Germany). cDNA was generated from 1μg RNA using iScript Advanced cDNA Synthesis Kit for RT-qPCR (170–8843; Bio-Rad). qPCR was run for ChREBP-α, ChREBP-β, L-PK, ACC, FAS, SREBP1, MondoA, SCD1, PEPCK, TNFα, IL1β, IL6, CD68 and 36B4 (reference gene) (sequences in Table 1) using custom oligonucleotides (MicroMon, Monash University) and SYBR Green PCR Master Mix (4309155, Life Technologies). Amplifications were performed using a Real Plex4 Mastercycler (Eppendorf, Germany) followed by a melt curve analysis.

**Table 1:** Primer sequences for RT-qPCR

Primer name	Primer sequence 5’– 3’ F	Primer sequence 5’– 3’ R
mChREBPα	CGACACTCACCCACCTCTTC	TTGTTCAGCCGGATCTTGTC
mChREBPβ	TCTGCAGATCGCGTGGAG	CTTGTCCCGGCATAGCAAC
mL-PK	CTTGCTCTACCGTGAGCCTC	ACCACAATCACCAGATCACC
mACC	GGACAGACTGATCGCAGAGAAAG	TGGAGAGCCCCACACACA
mSREBP1	AACGTCACTTCCAGCTAGAC	CCACTAAGGTGCCTACAGAGC
mMondoA	CAGCAGATCATCCACAGCGGCCAC	CTCGAAGAGCTTGGTGAGCGACGC
mSCD1	CCGGAGACCCTTAGATCGA	TAGCCTGTAAAAGATTTCTGCAAACC
mFAS	TTCCAAGACGAAAATGATGC	AATTGTGGGATCAGGAGAGC
mPEPCK	TGGCTACGTC CCTAAGGAA	GGTCCTCCAGATACTTGTCGA
mTNFα	CATCTTCTCAAAATTCGAGTGACAA	TGGGAGTAGACAAGGTACAACCC
mIL1β	CAACCAACAAGTGATATTCTCCATG	GATCCACACTCTCCAGCTGCA
mIL6	CACTTCACAAGTCGGAGGCTTA	GCAAGTGCATCATCGTTGTTC
mCD68	GCTACATGGCGGTGGAGTACAA	ATGATGAGAGGCAGCAAGATGG
h/m36B4	GCGACCTGGAAGTCCAACTAC	ATCTGCTGCATCTGCTTGG

### Statistical Analysis

**Fig 1:**
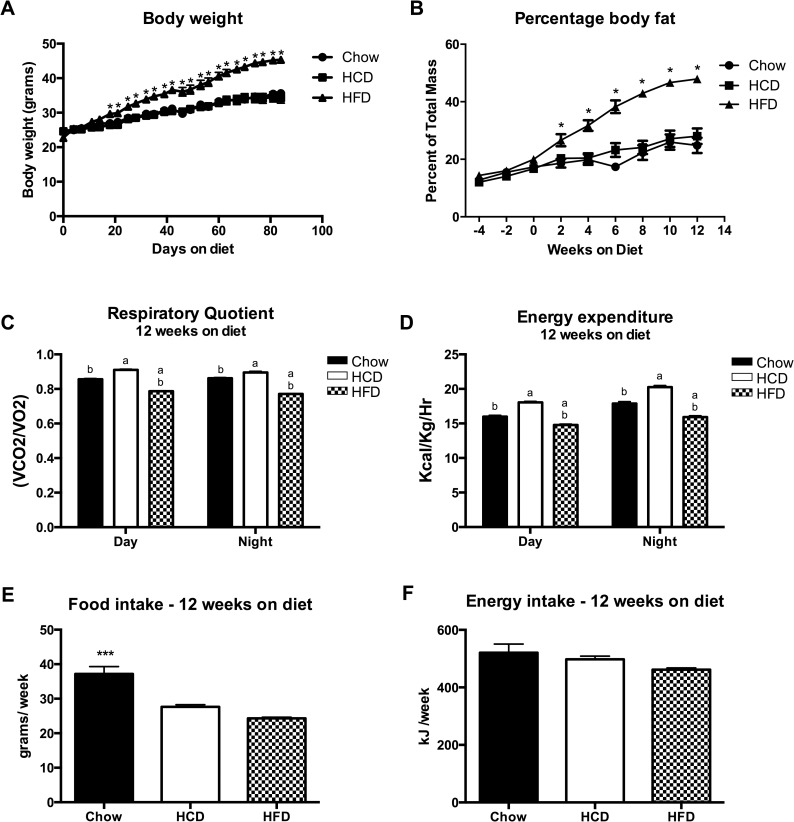
Characterisation of the effect of diet composition on C57BL/6 mice. (A) Effect of Chow, HCD and HFD on body weight over 12 weeks (n = 5 per group). (B) Effect of Chow, HCD and HFD on percentage body fat over 12 weeks, as assessed by DEXA scan (n = 5 per group). (C) RQ and (D) Energy expenditure from a representative day and night. Data from CLAMS metabolic cages after 12 weeks on diet (n = 4 per group). (E) Food intake and (F) Energy intake averaged over 12 weeks (n = 5 per group). Results are expressed as mean ± SEM. Statistical analysis was by two-way repeated measures ANOVA followed by Dunnett’s post-hoc test (A and B) or a one-way ANOVA followed by Dunnett’s post-hoc test (C, D and E). (*: p<0.05, ***: p<0.001) (a: p<0.05 vs Chow group, b: p<0.05 vs HCD group)

**Fig 2:**
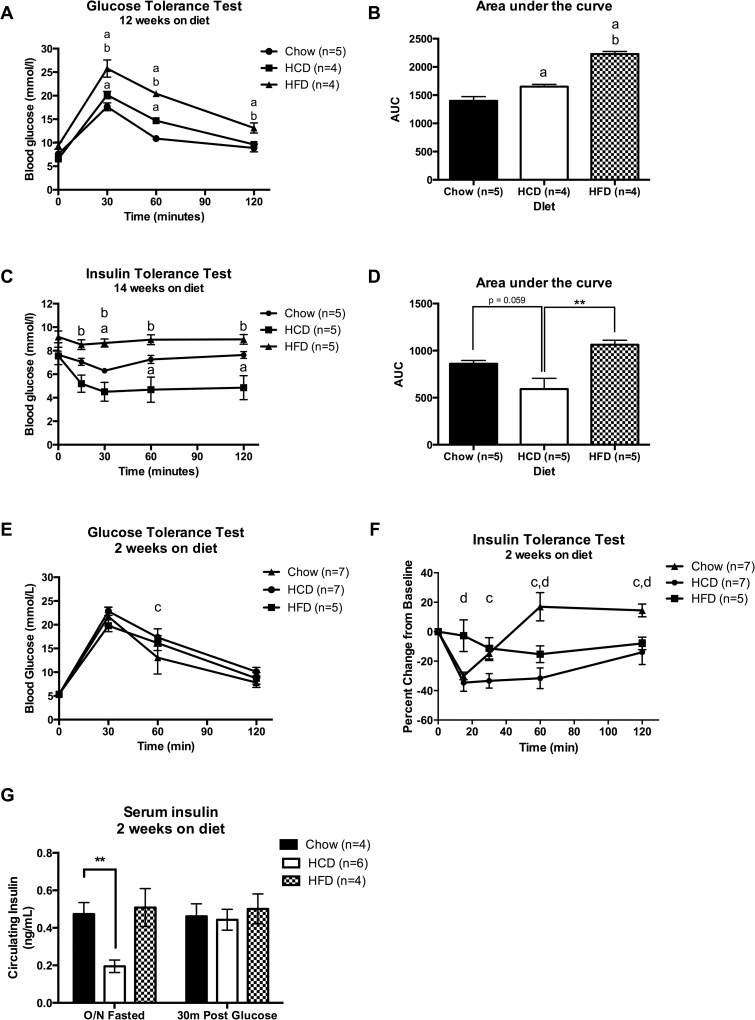
A high-calorie, high-carbohydrate diet results in glucose intolerance but improved insulin sensitivity. (A) Glucose tolerance test (GTT), (B) Area under the curve (AUC) of GTT, (C) Insulin tolerance test (ITT), and (D) AUC of ITT. Tests performed after 12–14 weeks on diet. (E) GTT and (F) ITT percentage change after 2 weeks on diet. (G) Serum insulin. Statistical analysis was by two-way repeated measures ANOVA followed by Dunnett’s post-hoc test (A, C, E and F), one-way ANOVA followed by Dunnett’s post-hoc test (B, D), or two-way ANOVA followed by Dunnett’s post-hoc test (G). (**: p<0.01, ***: p<0.001, ****: p<0.0001) (a: p<0.05 vs Chow group, b: p<0.05 vs HCD group, c: p<0.05 HCD vs chow, d: p<0.05 HFD vs chow)

**Fig 3:**
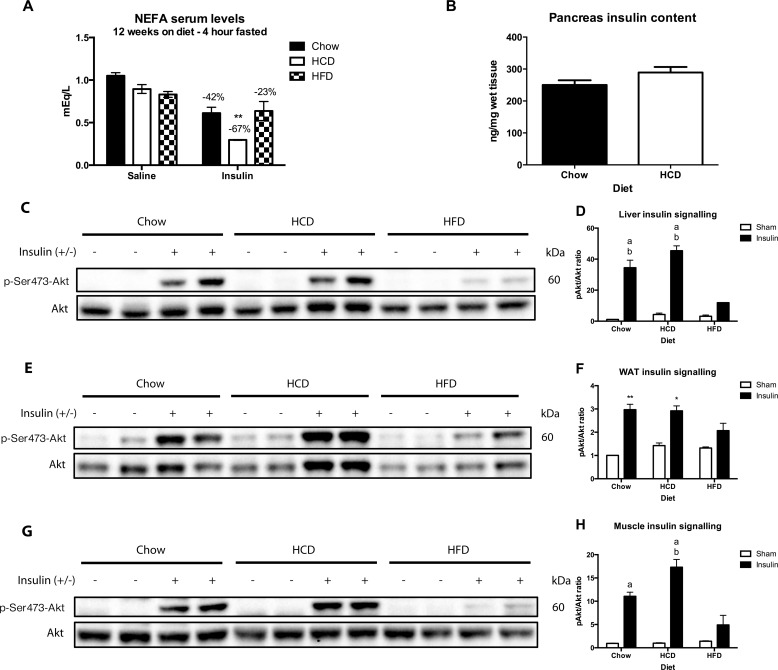
Effect of diet on sensitivity to exogenous insulin in C57BL/6 mice. (A) Serum non-esteried fatty acid (NEFA) levels 10 minutes after a saline or insulin injection (5U/mouse) (n = 3–4 per group). (B) Pancreatic insulin content after 12 weeks on diet (n = 13–14). Western blotting for Akt, p-Ser473-Akt in liver (C), WAT (E) and gastrocnemius muscle (G) of mice after a 4 hour fast followed by either a saline or insulin injection (5U/mouse) 10 minutes before sacrifice. Band density was analysed using Image J and pAkt/Akt ratio was calculated and graphed using chow/saline as control (D, F, H). Results are expressed as mean ± SEM. Statistical analysis was by two-way ANOVA followed by Dunnett’s post-hoc test (A, D, F, H). (*: p<0.05, **: p<0.01 vs sham controls) (a: p<0.01 vs sham controls, b: p<0.01 vs HFD insulin)

**Fig 4:**
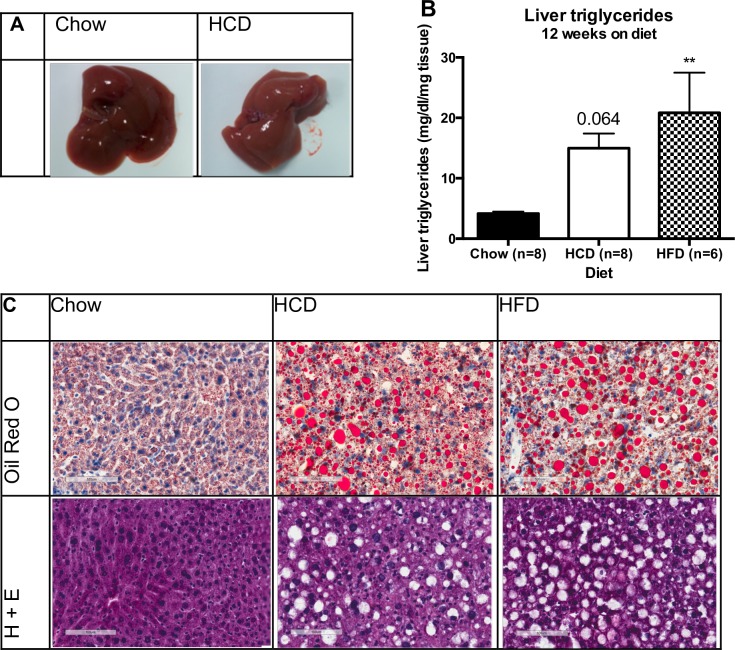
A high-calorie, high-carbohydrate diet results in liver steatosis. (A) A representative photo of a liver from mice fed a chow or a HCD. (B) Effect of diet on liver triglyceride levels in mice. (C) Representative Oil Red O and H+E liver sections from mice fed either a chow, HCD or HFD for 12 weeks. Magnification = 20x. Results are expressed as mean ± SEM. Statistical analysis was by one-way ANOVA followed by Dunnett’s post-hoc test (B). (**: p<0.01 vs chow mice)

**Fig 5:**
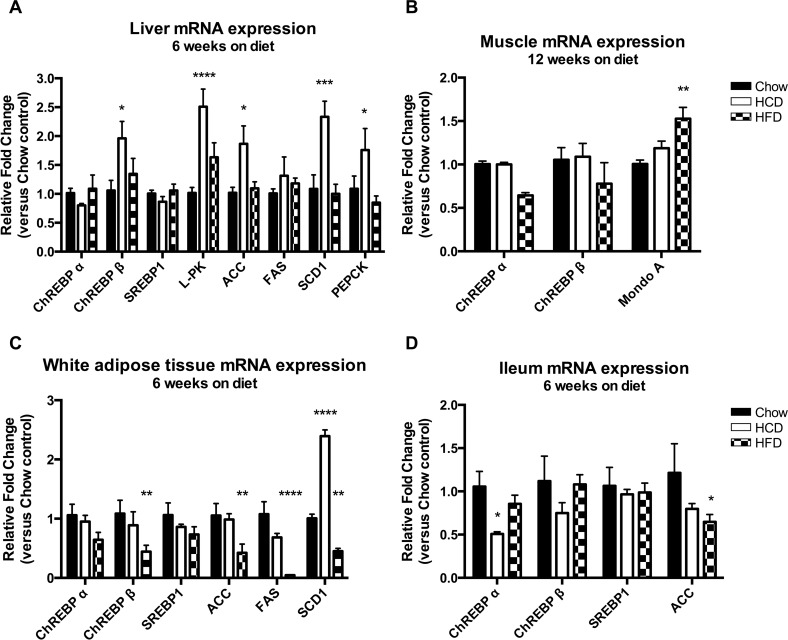
Effect of diet on ChREBP gene expression and the lipogenic pathway. Effect of diet on gene expression in liver (A), skeletal muscle (B), white adipose tissue (C) and ileum (D). N = 5 per group. Results were normalized to expression of the housekeeping gene 36B4, and then shown as fold change versus the chow control mice. Results are expressed as mean ± SEM. Statistical analysis was by two-way ANOVA followed by Dunnett’s post-hoc test. (*: p<0.05, **: p<0.01, ***: p<0.001, ****: p<0.0001 versus chow fed mice)

**Fig 6:**
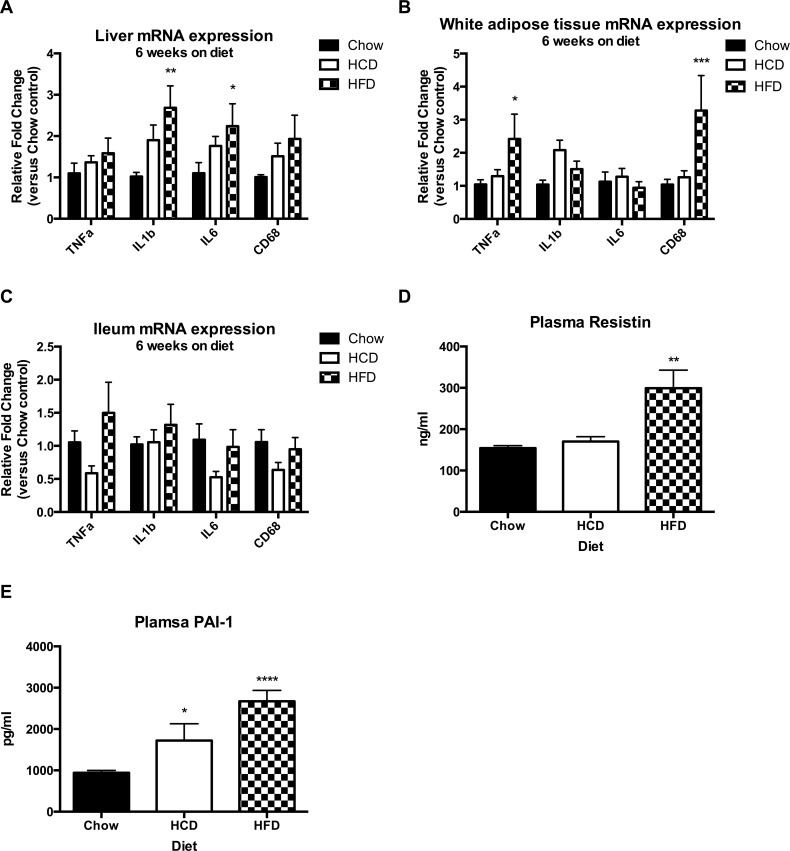
Effect of diet on inflammatory gene expression and serum inflammatory markers. Effect of diet on gene expression in liver (A), WAT (B) and ileum (C) and serum resistin (D) and PAI-1 (E). N = 5 per group. For gene expression, results were normalized to expression of the housekeeping gene 36B4, and then shown as fold change versus the chow control mice. Results are expressed as mean ± SEM. Statistical analysis was by two-way ANOVA (A, B, C) or one-way ANOVA (D, E) followed by Dunnett’s post-hoc test. (*: p<0.05, **: p<0.01, ***: p<0.001, ****: p<0.0001 versus chow fed mice)

All data are presented as mean ± SEM, with a statistically significant difference defined as p < 0.05. Data were analyzed by two-way repeated measures analysis of variance (ANOVA), two-way ANOVA or one-way ANOVA as indicated, followed by Dunnett’s post-hoc test. All graphs and statistical analyses were completed using GraphPad Prism (GraphPad Prism 6.0 for Mac OS X, GraphPad Software, Inc., San Diego, CA). See S1 Dataset for the raw data underlying Figs 1–6.

## Results

### A high calorie, high complex-carbohydrate diet does not cause weight gain in mice

Body weight of all mice was measured at 8 weeks of age, and then mice were randomly divided to receive a low-fat chow control diet (Chow, n = 5), a high-fat/high-calorie diet (HFD, n = 5), or a high-complex-carbohydrate/high-calorie diet (HCD, n = 5). Body weight was measured twice a week for 12 weeks (Fig 1A), and DEXA scanning by PIXImus was performed every 2 weeks to assess changes in body composition (Fig 1B). HFD-feeding resulted in increased body weight and percentage fat after approximately 2 weeks (p < 0.05), and this continued throughout the 12-week time period. HCD-feeding did not affect body weight or percentage fat when compared to the Chow-fed mice. There were no significant differences in bone mineral density or bone mineral content with any diet, as assessed by DEXA scanning (data not shown). Similarly, although mice fed chow showed increased food intake, there was no significant difference in energy intake between the groups (Fig 1E and 1F).

Mice were put into the Columbus Instruments Comprehensive Lab Animal Monitoring System (CLAMS) metabolic cages after 12 weeks on diet. Using these cages, oxygen consumption, carbon dioxide production and heat production were measured (thus allowing determination of respiratory quotient (RQ) and energy expenditure), as well as activity. As expected, the respiratory quotient was significantly higher in the HCD mice, and lower in the HFD mice, when compared to control Chow mice (Fig 1C) (p < 0.05). This is consistent with higher RQ with increased carbohydrate oxidation (towards 1), and lower RQ with increased fat oxidation (towards 0.7). This was accompanied by increased energy expenditure in the HCD mice, and lower energy expenditure in the HFD mice, when compared to control Chow mice (Fig 1D) (p < 0.05). There were no significant changes in activity levels between the groups (data not shown).

### HCD-fed mice have increased sensitivity to exogenous insulin, but impaired glucose tolerance

To assess whether HFD-feeding or HCD-feeding altered the ability to handle a glucose load, a glucose tolerance test (GTT) was performed after 12 weeks on diet (Fig 2A). Furthermore, an insulin tolerance test (ITT) was performed after 14 weeks on diet to assess the effect of diet on peripheral insulin sensitivity (Fig 2C). HFD-fed mice showed impaired ability to clear glucose during the GTT, and this resulted in an increased area under the curve (AUC) when compared to both the HCD-fed mice and Chow-fed mice, suggesting they were glucose intolerant (Fig 2B) (p < 0.05). HCD-fed mice have improved glucose tolerance when compared to HFD-fed mice, however they still showed modest glucose intolerance, with slower glucose clearance and an increased AUC when compared to Chow-fed mice (Fig 2C) (p < 0.05). Despite this reduced glucose tolerance, HCD-fed mice showed enhanced insulin sensitivity when compared to both the HFD-fed and Chow-fed mice, as shown by response to insulin in the ITT (Fig 2C) (p < 0.05). This enhanced peripheral insulin sensitivity in response to HCD feeding was unexpected, and in order to tease out when this phenotype develops another cohort of mice was put on diet and ITTs and GTTs were performed every 2 weeks. After only two weeks of diet HCD-fed mice showed enhanced peripheral insulin sensitivity (Fig 2F) (p < 0.05) and mild glucose intolerance (Fig 2E) (p < 0.01) when compared to Chow-fed mice.

HCD-fed mice had lower overnight fasted circulating insulin levels after two weeks on diet, but no difference 30 minutes after a glucose bolus (Fig 2G) (p < 0.01). Furthermore, there was no significant difference in pancreatic insulin content between Chow and HCD fed mice after 12 weeks on diet (Fig 3B). However, after 12 weeks on diet exogenous insulin administration resulted in a profound decrease in non-esterified fatty acids (NEFAs) in HCD-fed mice (67% reduction compared to saline control), which was significantly larger than both Chow-fed mice (42% reduction) and HFD-fed mice (23% reduction) (Fig 3A) (p < 0.01). This once again highlights the enhanced insulin sensitivity of mice fed a HCD.

### HCD-feeding increases hepatic steatosis in mice

Upon study completion, macroscopic examination of the livers of all mice highlighted that HCD-fed mice had significantly paler livers when compared to Chow-fed mice, suggesting this diet may be causing liver steatosis (Fig 4A). Assessment of hepatic triglyceride levels confirmed that HFD-fed mice had significantly higher liver triglycerides compared to Chow-fed mice (Fig 4B) (p < 0.01) but HCD-fed mice only showed a trend towards higher liver triglyceride content, which did not reach statistical significance (p = 0.06). However, Oil Red-O staining of mouse livers revealed a clear increase in liver steatosis in both HFD-fed and HCD-fed mice (Fig 4C). Interestingly, this increase in liver steatosis in HCD-fed mice occurred despite no changes in body weight or percentage body fat (Fig 1A and 1B), and did not seem to affect liver insulin sensitivity, as discussed below.

### Insulin sensitivity was maintained in peripheral tissues from HCD-fed mice

In order to determine insulin sensitivity in key metabolic tissues, mice were injected with either insulin or saline and pAkt/Akt protein concentration was determined via Western Blotting in liver, white adipose tissue (WAT) and muscle (gastrocnemius) (Fig 3). As expected, HFD-fed mice had reduced pAkt in response to insulin in muscle, adipose and liver when compared to both Chow and HCD fed mice, suggesting insulin resistance in all three tissues. In contrast, HCD-fed mice showed enhanced pAkt in response to insulin (Fig 3C, 3D and 3E), as well as a trend towards enhanced pAkt/Akt ratio in response to insulin in muscle and liver, and comparable levels in adipose tissue when compared to Chow-fed mice (Fig 3). HCD-fed mice have enhanced whole body insulin sensitivity, and this is paralleled with enhanced insulin sensitivity in peripheral tissues.

### The composition of diet influences the expression of ChREBP and its lipogenic target genes in a tissue-specific manner

The increased triglyceride content in the livers of HCD-fed mice, despite no changes in body composition or fat intake, suggests there might be an increase in liver de novo lipogenesis (DNL). ChREBP and SREBP1 are two lipogenic transcription factors, and activate transcription of genes involved in DNL. For this analysis we compared the mRNA levels of ChREBPα, ChREBPβ, SREBP1, L-PK and ACC in liver, WAT and ileum for all groups. In liver, HCD feeding resulted in increased mRNA expression of ChREBPβ (≈2-fold versus Chow), but not ChREBPα, with a concordant increase in the ChREBP target genes L-PK (2.5-fold) and ACC (1.9-fold), but not FAS (Fig 5A) (p < 0.01). Furthermore, HCD feeding resulted in increased expression of SCD1, where it was approximately 2.3 fold higher than both HFD and Chow fed mice (Fig 5A) (p < 0.0001). Interestingly, HCD feeding also increased hepatic PEPCK expression suggesting elevated hepatic glucose production in these mice (1.8-fold) (Fig 5A) (p<0.05). In contrast, HFD-feeding resulted in decreased mRNA expression of ChREBPβ in WAT (≈0.4-fold) (p < 0.05), with a trend towards decreased ChREBPα (0.6-fold) (p = 0.06), along with decreased expression of ACC (≈0.4-fold), FAS (0.05-fold) and SCD1 (0.5-fold) (Fig 5C) (p < 0.01). Although HCD feeding did not alter mRNA expression of ChREBP or SREBP1 there was a robust increase in SCD1 in WAT (2.4-fold) (Fig 5C) (p<0.0001). In ileum, there was a decrease in ChREBPα with HCD feeding, but this was not accompanied by any changes in ChREBPβ or lipogenic target genes (Fig 5D) (p < 0.05). There were no significant changes in ChREBP expression in muscle, although there was a slight increase in MondoA in HFD fed mice (1.5-fold) (Fig 5E) (p<0.01). These results reveal that not only is ChREBP expression influenced by the composition of diet in a tissue-specific manner, but also that there is a differential effect on the two ChREBP isoforms; ChREBPα and ChREBPβ.

### HFD, but not HCD, feeding increases markers of inflammation in mice

Recent studies have shown ChREBP may have important anti-inflammatory effects. To investigate whether the diet-induced changes in ChREBP associated with diet-induced inflammation in mice, inflammatory mRNA expression was evaluated in liver, WAT and ileum, as well as the serum levels of inflammatory markers resistin and PAI-1. In liver, HFD-fed mice had increased levels of IL1β and IL6 mRNA when compared to chow-fed mice (Fig 6A) (p < 0.05). Interestingly, this was not the case for HCD-fed mice, which showed no significant difference to chow-fed mice despite presenting with hepatic steatosis. In WAT, HFD-feeding resulted in increased TNFα mRNA expression, as well as increased expression of the macrophage marker CD68 (Fig 6B) (p < 0.05). There were no significant effects of diet on inflammatory gene expression in the ileum (Fig 6C). HFD fed mice had increased plasma levels of resistin and PAI-1 when compared to chow-fed mice, whereas HCD-fed mice showed elevated levels of PAI-1 but not resistin (Fig 6D and 6E) (p < 0.05). These results show an association between a reduction in adipose ChREBP and an increased inflammatory burden with HFD feeding.

## Discussion

The *de novo* lipogenic transcription factor ChREBP has been implicated in various metabolic disorders, including non-alcoholic fatty liver disease and insulin resistance [6, 14, 18, 19, 35–37]. It is a glucose-responsive transcription factor, and as such is sensitive to changes in the composition of diet. In this study, we sought to determine the differential effects of a high calorie, high complex-carbohydrate diet (HCD), a high calorie, high-fat diet (HFD), and a low calorie, low-fat chow diet (Chow) on the metabolic function and ChREBP expression in C57BL/6 mice. Such dietary manipulations allowed us to assess the effect of a diet of equal caloric content in the absence of weight gain seen with a HFD. Furthermore, although various studies have investigated the effects of high-carbohydrate administration on ChREBP expression, these diets have been high in sugars [23–25]. An important point in this study is that we examined the effects of two high-caloric diets with equal amount of sucrose that differed in fat and complex-carbohydrate only. The HCD mice displayed mild glucose intolerance and increased insulin sensitivity in comparison to glucose intolerance and insulin resistance when mice were placed on a HFD. Both diets resulted in hepatic steatosis yet had dramatically different effects on insulin signalling in liver, WAT and muscle. As expected, we found the composition of diet differentially regulated the expression of ChREBP isoforms in different tissues. The HCD-fed mice had a selective increase in the expression of ChREBPβ in liver, however no significant changes in ChREBP expression were observed in adipose tissue or muscle. Conversely, the HFD mice showed a robust decrease in adipose tissue ChREBP expression along with lipogenic target genes. Interestingly, these changes in ChREBP expression in adipose were inversely associated with markers of inflammation, suggesting ChREBP may be involved in diet-induced inflammation in this tissue.

Mice fed a HFD gained weight and body fat as expected, however there was no difference between the HCD-fed and regular Chow-fed mice (Fig 1A and 1B). Analysis of metabolic respiratory parameters confirmed the macronutrient intake, with expected higher RQ for HCD-fed mice and lower RQ for HFD-fed mice (Fig 1C). Importantly, there were similar changes in energy expenditure (EE), with HCD-fed mice having significantly higher EE and HFD-fed mice having significantly lower EE (Fig 1D). This is in agreement with previous studies showing high carbohydrate feeding increases energy expenditure, presumably through increases in the thermic effect of food, in both human and animal models [37, 38]. Mechanistically, this may be due to increased expression and activity of uncoupling proteins, which increase thermogenesis and decrease the efficiency of energy use [39], and could explain why HCD-fed mice do not gain weight.

HFD-fed mice had impaired glucose tolerance and insulin sensitivity after 12 weeks on diet as assessed by tolerance tests, and impaired sensitivity to exogenous insulin in liver, muscle and adipose as assessed by pAkt signalling (Fig 2A and 2C and Fig 3). However, unexpectedly the HCD-fed mice showed slightly impaired glucose tolerance and significantly enhanced sensitivity to exogenous insulin when compared to Chow-fed mice and this was without any apparent changes in body composition (Fig 2A and 2C and Fig 1A and 1B). This was seen across multiple tests, such as a physiological insulin tolerance test and biochemically in muscle, adipose and liver where administration of exogenous insulin resulted in enhanced pAkt signalling in HCD-fed mice (Fig 3). The enhancement in insulin sensitivity from a HCD was evident after only two weeks of feeding, and occurred despite an increase in total liver triglyceride levels. The increased hepatic steatosis in response to HCD-feeding, as shown by Oil-Red-O staining, was associated with an increased expression of hepatic ChREBPβ and surprisingly had no effect on liver insulin signalling (Fig 3C and 3D). This is contradictory to human studies that show an association between hepatic ChREBP and insulin resistance in obesity [6, 14], and suggests this increase is compensatory and may even be protective in defending against substrate overload. The fact that there was no change in SREBP1 mRNA expression highlights that ChREBP is primarily responsible for the increase in liver triglycerides in response to a HCD, although assessing the nuclear content of each transcription factor will be important in confirming this.

An increase in liver triglycerides has been linked to insulin resistance [40], however recent studies have postulated that it is particular lipid intermediaries that are involved in lipotoxicity, and increasing *de novo* lipogenesis may in fact alleviate insulin resistance by removing these harmful lipids [18, 41, 42]. In fact, in a study by Benhammed et al., adenoviral mediated over-expression of ChREBP resulted in profound hepatic steatosis in mice, but improved insulin sensitivity under HFD-feeding conditions [18]. The authors proposed this was due to increased expression of SCD1 resulting in conversion of lipotoxic, saturated fatty acids to more beneficial monounsaturated fatty acids. Indeed, in our study increased expression of ChREBPβ in liver was associated with increased expression of SCD1 in HCD-fed mice, and this was not seen in HFD-fed or Chow-fed mice (Fig 5A). A novel finding of this study is that HCD-fed mice also had increased SCD1 expression in adipose tissue suggesting increased *de novo* lipogenesis in this tissue as well (Fig 5C). This could provide a mechanistic basis for the enhanced insulin sensitivity seen with this diet. Previous studies have shown adipose produced palmitoleate, which is synthesized by SCD1, can signal to liver and muscle to enhance insulin signalling in mice [43]. The enhanced *de novo* lipogenesis and subsequent increased production of insulin-sensitizing lipids induced by HCD feeding may be responsible for the enhanced insulin sensitivity seen in these mice.

ChREBP has also been shown to synthesize a novel class of insulin sensitizing and anti-inflammatory lipids, called branched fatty acid esters of hydroxy fatty acids (FAHFAs), which are present in brown and white adipose tissues, liver and serum [37]. Further lipidomic studies examining the particular lipid species present in liver and adipose tissue in response to HCD and HFD may help explain the differing metabolic effects. Interestingly, along with impaired insulin signalling in liver, HFD-fed mice displayed increased mRNA expression of the inflammatory markers IL1β and IL6, which was not seen in HCD-fed mice (Fig 6A). Therefore, although both HCD-fed and HFD-fed mice displayed hepatic steatosis, the difference in insulin sensitivity may be due to HCD preferentially inducing ChREBP, resulting in production of anti-inflammatory insulin-sensitizing lipids. Increased markers of inflammation were also evident in WAT of HFD-fed mice, with increased mRNA expression of the inflammatory cytokine TNFα as well as the macrophage marker CD68 (Fig 6B). These changes were associated with a robust reduction in ChREBP and its target genes, whereas HCD-feeding did not reduce ChREBP and did not increase markers of inflammation. The adipokines resistin and PAI-1 were also increased in serum of HFD-fed mice, further suggesting a state of chronic inflammation.

The reduction in WAT ChREBP expression in response to HFD-feeding has been shown previously [25], and fits well with human studies showing reduced adipose ChREBP in obesity and type 2 diabetes [14, 15]. However, the association with inflammation suggests a novel role for ChREBP in diet-induced inflammation. *Nuotio-Antar et al*. genetically over-expressed ChREBP in adipose tissue of mice, which decreased adipose inflammatory gene expression in response Western diet, thus supporting an anti-inflammatory role for ChREBP [44]. However, whether these effects were due to ChREBP action in adipocytes or macrophages is unclear. Importantly, ChREBP is reduced rapidly in response to HFD-feeding suggesting it may be an early contributor to the development of insulin resistance [25]. This may be due to a reduction in novel anti-inflammatory insulin-sensitizing lipids as outlined above. Alternatively, ChREBP has recently been shown to play an important role in redox balance in macrophages. ChREBP inhibits reactive oxygen species accumulation, macrophage M1 polarisation and inflammatory responses, and this has been shown to be important in atherosclerotic plaque development in mice [45]. Whether ChREBP plays a similar role in adipose tissue macrophages in diet-induced inflammation and obesity is unknown and will require future investigation. If it does, the subsequent altered pro-inflammatory adipokine profile may contribute to the development of insulin resistance and diabetes in obesity. Interestingly, *Perry et al*. have shown adipose tissue inflammation due to high-fat feeding results in inappropriately upregulated lipolysis, which prevents insulin’s suppression of hepatic glucose production by increasing WAT-derived hepatic acetyl-coA and therefore increasing pyruvate carboxylase activity [46]. This provides a link between adipose inflammation and hepatic insulin resistance and may be an important cause of the detrimental hyperglycemia seen in obesity and type 2 diabetes. Whether ChREBP down-regulation in adipose tissue macrophages is an early contributor to this will be an interesting question to address.

Despite improvements in insulin sensitivity, mice fed a HCD have a moderate impairment in the ability to dispose of a glucose load. This correlates with increased expression of the rate-controlling gluconeogenic enzyme PEPCK. This glucose intolerance may be due to carbohydrate overload interfering with appropriate glucose responses. In a hepatoma cell line, increasing media glucose concentration for an extended period of time results in attenuated glucose and insulin inhibition of PEPCK expression and enhanced cAMP stimulated PEPCK expression [47]. Although our HCD is not high in sugars, the overall increase in carbohydrate load may result in a prolonged glucose delivery and interfere with normal suppression of hepatic glucose production. This was unexpected as *in vitro* studies have shown ChREBP can bind the PEPCK gene and repress its transcription [48], so with the increase in hepatic ChREBP a decrease in PEPCK expression was expected. These conflicting results may be due to the differences between *in vitro* and *in vivo* models and highlights the complexity of whole body glucose metabolism. The paradoxical increase in hepatic glucose production despite increased serum insulin has been shown in healthy humans fed high dietary carbohydrate [49]. This response is most likely protective in the short-term, and has been suggested to help protect against accumulation of phosphorylated intermediates [50]. However, in the long-term this may result in hyperglycemia and eventual progression to diabetes. As these mice were only on diet for 12 weeks we cannot conclude what chronic effects this diet may have. However, the influence of carbohydrate overload may be especially important in the pathogenesis of diabetes in populations that primarily consume refined complex carbohydrates for energy.

Our data provide further support for a feed-forward model of ChREBP regulation where ChREBPα binds to the carbohydrate response element on the ChREBP gene promoter and activates the transcription of the more active ChREBPβ [6]. We found in both liver and adipose tissue that ChREBP target gene expression followed ChREBPβ expression, not ChREBPα. Furthermore, ChREBPβ mRNA expression, and not ChREBPα, was robustly regulated by dietary manipulations. This suggests transcription of ChREBPβ is an important regulatory mechanism, and supports measurement of ChREBPβ mRNA expression as an indicator of ChREBP activity. This study has confirmed that ChREBP isoforms are differentially regulated by diet in a tissue-specific manner and show that macronutrient composition has profound effects on ChREBP expression regardless of caloric intake. Furthermore, we have described the selective upregulation of hepatic ChREBPβ in response to a diet high in complex carbohydrates along with the associated complex metabolic profile.

In conclusion, we have shown ChREBPβ selectively responds to macronutrient composition in a tissue specific manner. Although an increase in hepatic ChREBPβ associates with hepatic steatosis, this is not associated with insulin resistance. A reduction in ChREBPβ does associate with insulin resistance as well as inflammation in metabolic tissues. The importance of ChREBP down-regulation in obesity induced inflammation and its role in the development of insulin resistance and type 2 diabetes will be important to investigate.

## Supporting Information

S1 Dataset Raw data underlying Figs 1–6. The raw data used for Figs 1–6.
(XLSX)

S1 File Diet compositions. Detailed diet compositions for high-fat, high-carbohydrate and low-fat chow diets used in this study.
(XLSX)
